# How Can Maternal Lifestyle Interventions Modify the Effects of Gestational Diabetes in the Neonate and the Offspring? A Systematic Review of Meta-Analyses

**DOI:** 10.3390/nu12020353

**Published:** 2020-01-29

**Authors:** Delphine Mitanchez, Cécile Ciangura, Sophie Jacqueminet

**Affiliations:** 1 Department of Neonatology, Bretonneau Hospital, François Rabelais University, F-37000 Tours, France; 2 INSERM UMR_S 938 Centre de Recherche Saint Antoine, F-75012 Paris, France; 3 Department of Diabetology, Institute of Cardiometabolism and Nutrition (ICAN), APHP, University Hospital Pitié-Salpêtrière, F-75013 Paris, France; cecile.ciangura@aphp.fr (C.C.); sophie.jacqueminet@aphp.fr (S.J.)

**Keywords:** diabetes, obesity, gestational weight gain, macrosomia, adiposity, neonate, diet, exercise

## Abstract

Gestational diabetes (GDM) has deleterious effects on the offspring. Maternal obesity and excessive gestational weight gain (GWG), often associated with diabetes, also contribute to these adverse outcomes. Objectives: To assess the benefit for the offspring of maternal lifestyle interventions, including diets and physical activity, to prevent or to improve GDM and to limit excessive GWG. Method: Systematic review of meta-analyses published in English between December 2014 and November 2019. Results: Lifestyle interventions to reduce the risk of GDM reported a decreased risk of 15% to 40%, with a greater effect of exercise compared to diet. Combined lifestyle interventions specifically designed to limit GWG reduced GWG by 1.6 kg in overweight and obese women, and on average by 0.7 to 1 kg in all pregnant women. In these trials, adverse neonatal outcomes were poorly studied. Combined lifestyle interventions in women with GDM significantly reduced fetal growth. Altogether, lifestyle interventions reduced the risk of preterm birth and shoulder dystocia, but individually, diets or exercise alone had no effect on neonatal adverse outcomes. Conclusion: Specific maternal, neonatal and offspring benefits of lifestyle interventions during pregnancy to prevent or improve GDM control or to limit GWG still require clarification.

## 1. Introduction

Gestational diabetes mellitus (GDM) is defined as carbohydrate intolerance of variable severity first recognized during pregnancy. However, this definition encompasses two different entities: a glucose tolerance defect which generally occurs in the second half of pregnancy and then disappears in the post-partum period and overt diabetes in pregnancy (DIP) which is undiagnosed prior to the beginning of pregnancy, mainly as type 2 diabetes. Recently, the American Diabetes Association proposed a clearer definition of GDM: “diabetes diagnosed in the second or third trimester of pregnancy that was not clearly overt diabetes prior to gestation” [1].

A recent meta-analysis confirmed the evidence on the relationship between an increased pre-pregnancy body mass index (BMI) and the risk of GDM. It showed that the GDM risk increased 4% per unit of increase in BMI [2]. In an individual participant data meta-analysis of over 265,000 births, the risk of BMI per kg/m^2^ was 1.12 (95% CI 1.12–1.13) and the highest risk was for grade 3 obesity (BMI ≥ 40 kg/m^2^) with an OR 7.59 (95% CI 6.14, 9.38). It was estimated that 42.8% of GDM cases were weight gain (GWG) was associated with increased risk of GDM (per SD increase in GWG, OR 1.14, 95% CI 1.10, 1.18) and obese mothers with high GWG were at the highest risk [3].

Maternal hyperglycemia during pregnancy has deleterious effects at all stages of fetal development. A large body of evidence support the association between pregestational diabetes and increased risk of malformations [4]. In the fetus, maternal diabetes leads to fetal hyperinsulinemia, the direct consequence of which is high birth weight and increased fat mass [5,6]. Fetal overgrowth exposes the fetus to an increased risk of a number of neonatal complications, including asphyxia, obstetrical trauma, respiratory distress and hypoglycemia. The risk of these different complications is higher in pre-gestational diabetes compared to GDM [7].

Maternal obesity and excessive GWG, often associated with diabetes, also contribute to these adverse outcomes [8]. In addition, alteration of the intrauterine environment secondary to maternal diabetes, obesity and excessive GWG contributes to the early determinism of adult diseases in offspring, including obesity, diabetes and metabolic syndrome [8,9].

Many studies have investigated the effect of lifestyle interventions, mainly including exercise and diets, on maternal glycemic and GWG control. This is a systematic review exploring data from meta-analyses in order to evaluate: (1) the benefit of lifestyle interventions to prevent GDM and limit pre-pregnancy and gestational weight gain for the offspring and (2) the effectiveness of lifestyle interventions in women with GDM to improve health outcome in the offspring.

## 2. Methods

The MEDLINE database was systematically reviewed for papers on human subjects published in English between December 2014 and November 2019. We used the MeSH terms diabetes, gestational, obesity, pregnancy, gestational weight gain, treatment, neonate, and offspring. For this review, we selected meta-analyses that addressed lifestyle interventions for the prevention of GDM or for the improvement of pregnancy outcomes in the case of GDM and meta-analyses that specifically addressed measures to limit GWG. We also looked at meta-analyses that addressed the effect of bariatric surgery on pregnancy outcomes. Only analyses that included more than two trials were recorded.

**Figure 1:**
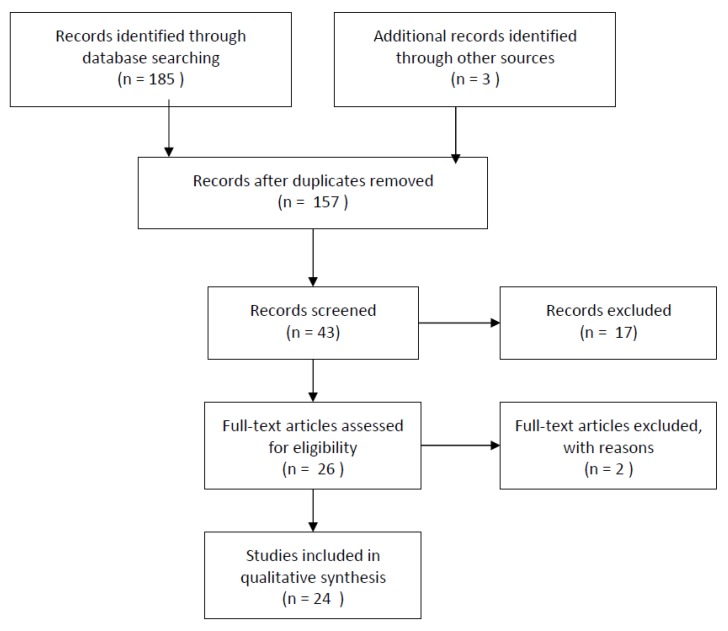
Flow diagram.

One hundred and eighty-eight records were identified and there were 157 records after exclusion of duplicates. Forty-three were screened and 26 full-text articles were assessed for eligibility. Two were excluded: one because it was a Cochrane analysis from 2015 updated in 2017, and the other because two records were based on the same data (Figure 1).

Macrosomia was defined as birth weight > 4000 g, unless another definition is clarified. Large-for-gestational age (LGA) was defined as birth weight > 90th percentile, based on sex and gestational age. Small-for-gestational age (SGA) was defined as birth weight < 10th percentile.

## 3. Results

### 3.1. Lifestyle Interventions to Prevent GDM and Maternal Weight Gain

This chapter explores the extent to which lifestyle interventions reduce the risk of GDM, or excessive maternal weight gain during pregnancy and the consequences on the health of the newborn and the child based on data from selected meta-analysis. Similarly, it explores the effects of pre-gestational weight loss observed with bariatric surgery on the health of the offspring.

#### 3.1.1. Lifestyle Interventions to Prevent GDM (Table 1)

In a systematic review and meta-analysis of randomized clinical trials (RCT) including 11,487 pregnant women, Song et al. assessed the effect of lifestyle interventions during pregnancy, including diet and physical activity or both, on the risk of GDM. An 18% (95% confidence interval (CI) 5, to 30%; *p* = 0.0091) reduction in the risk of GDM was reported in that study. When combined diet and exercise, diet alone, and exercise alone interventions were considered separately, the observed reductions in GDM were no longer statistically significant, although the direction of effect for each type of intervention did suggest a benefit. Furthermore, subgroup analysis showed that such an intervention was effective only if initiated before the 15th gestational week (relative risk: 0.80, 95% CI 0.66, 0.97), but not among women receiving the intervention afterwards. The effect size was similar among women with pregnancy overweight or obesity (RR 0.83, 95% CI 0.69, 1.00) [10].

In an updated Cochrane review, including 23 RCTs involving almost 9000 women, Shepherd et al. compared combined diet and exercise interventions to no interventions (standard care) in pregnant women for preventing GDM. In the group with interventions, there was a reduced risk of GDM (RR 0.85, 95% CI 0.71, 1.01, *p* = 0.07), and a reduction in GWG (−0.89 kg, 95% CI −1.39, −0.40). Among infants born to mothers receiving interventions, there was no difference between groups for perinatal mortality (RR 0.82, 95% CI 0.42, 1.63), for LGA (RR 0.93 95% CI 0.81, 1.07), neonatal hypoglycemia (RR 1.42 95% CI 0.67, 2.98), or other adverse neonatal outcomes, except for macrosomia (birth weight > 4500 g) (RR 0.63, 95% CI 0.42, 0.94), preterm birth (RR 0.80, 95% CI 0.65, 0.98) and respiratory distress syndrome (RR 0.56, 95% CI 0.33, 0.97) [11]. Subgroup analyses revealed no clear differential treatment effects according to maternal BMI. In this review, the authors were unable to consider comparisons of different types of combined diet and exercise interventions. In addition, they analyzed childhood outcomes and showed that diet and exercise had no effect on weight and adiposity in infancy, nor on blood pressure, fasting glucose and insulin, HDL, triglycerides and metabolic syndrome.

Guo et al. examined the effectiveness of lifestyle interventions, including diet, exercise or both, on GDM prevention and aimed at identifying key effectiveness moderators to improve the prevention strategy. They showed that diet and exercise during pregnancy were preventive of GDM (RR 0.77, 95% CI 0.69, 0.87, *p* < 10^−3^). Four key aspects were identified to improve the preventive effect: targeting the high-risk population, an early initiation of the intervention, the correct intensity and frequency of exercise and GWG management. In overweight or obese women, BMI failed to predict the effectiveness of an intervention. Instead, interventions were most effective in populations with high incidence of GDM rather than simply in women who are overweight or obese. Furthermore, moderate intensity exercise for 50–60 min twice a week could lead to an approximately 24% reduction in GDM [12].

Tieu et al. assessed the effects of dietary advice interventions for preventing GDM. A trend towards a reduction in GDM (RR 0.60, 95% CI 0.35, 1.04; *p* = 0.07) and lower GWG (−4.70 kg, 95% CI −8.07, −1.34) was observed for women receiving dietary advice compared with standard care. Subgroup analysis suggested a greater effect on GDM incidence for overweight and obese women receiving dietary advice (RR 0.39, 95% CI 0.19, 0.79). There were no clear differences between the dietary advice intervention and standard care groups for growth at birth, adverse neonatal outcomes and body weight, adiposity and blood pressure at six months of life [13].

Two studies assessed the effectiveness of physical exercise interventions during pregnancy to prevent GDM and excessive maternal weight gain. Sanabria-Martinez et al. found, in a sample of 2873 women, that physical exercise programs during pregnancy decreased the risk of GDM (RR = 0.69; 95% CI 0.52, 0.91; *p* = 0.009), particularly when the exercise program was performed throughout pregnancy starting from the first trimester (RR = 0.64; 95% CI 0.36, 0.98; *p* = 0.038). A decrease in maternal weight was also observed. (−1.14 kg; 95% CI −1.50, −0.78; *p* < 0.001). There was a wide variety in the type of exercises, frequency and intensity level and in most of the studies included, the adherence rate was high (>85%) [14]. Russo et al. reported a 28% reduction in the risk of GDM in the intervention group, in a meta-analysis involving 3401 participants (RR 0.72 95% CI 0.58, 0.91; *p* = 0.005). All of the interventions included at least an aerobic component (walking, land or water aerobics or both, cycling) and when reported, adherence in the intervention arm varied from 16.3% to greater than 95% [15]. In both studies, analyses were not performed according to maternal BMI and the outcome of the offspring was not studied.

Ming et al. investigated the effect of exercise during pregnancy on the occurrence of GDM among 3256 normal-weight pregnant women. The majority of the interventions adopted comprehensive exercise programs of light to moderate intensity that were performed three times per week. Exercise during pregnancy was shown to decrease the occurrence of GDM (RR = 0.58, 95% CI 0.37, 0.90; *p* = 0.01) and GWG (−1.61, 95% CI −1.99, −1.22; *p* < 0.01), but had no significant effect on gestational age at birth and birth weight [16].

In a systematic review, Davenport et al. reported that prenatal exercise-only interventions, but not exercise with co-interventions (e.g., dietary intervention), reduced the odds of GDM (n = 6934, OR 0.62, 95% CI 0.52, 0.75) [17]. One study conducted a meta-analysis of 16 cohort studies containing information on physical activity either prior to or at the commencement of pregnancy to prevent GDM. Compared to no physical activity, any pre-pregnancy or early pregnancy physical activity was associated with a 30% (OR = 0.70, 95% CI 0.57, 0.85; *p* < 10^−3^) and a 21% (OR = 0.79, 95% CI 0.64, 0.97; *p* = 0.03) reduced risk of GDM, respectively. Engaging in >90 min/week of leisure time physical activity before pregnancy was associated with a 46% decreased risk of GDM (OR = 0.54, 95% CI 0.34, 0.87; *p* = 0.01) [18]. These two studies did not report on neonatal outcome.

The results of these studies are summarized in Table 1.

#### 3.1.2. Lifestyle Interventions to Control Pregnancy Weight Gain (Table 2)

Data from seven clinical centers that conducted separate RCT to test different lifestyle intervention strategies to modify GWG were combined to conduct an individual-participant data (IPD) meta-analysis. This included 1150 women with BMI ≥ 25 kg/m^2^ randomized either to the group with interventions targeting diet, physical activity, and behavioral strategies or to the group with standard of care [19]. Mean total GWG was 1.6 kg less for the intervention group (−1.58, 95% CI −2.18, −0.99; *p* < 10^−3^) and the percentage of women with GWG per week below Institute of Medicine (IOM) guidelines was significantly higher in the intervention group than the standard care group (20.6% vs. 14.2%; *p* = 0.002, OR 1.65, 95% CI 1.20, 2.27). There was no difference between groups for the incidence of GDM (OR 0.92, 95 CI 0.61, 1.40). Preterm birth prior to 37 and 32 weeks did not differ by group. However, preterm birth prior to 28 weeks was significantly lower in the intervention group (OR 0.46, 95% CI 0.22, 0.95; *p* = 0.037). There was no difference for growth at birth (birth weight, LGA, SGA) or for adverse neonatal outcomes (malformations, death, respiratory morbidity, hypoglycemia) [19].

In a meta-analysis of RCT including overweight and obese women, Shieh et al. found that heathy eating (specified calorie and macronutrient goals and healthy eating strategies) and/or physical activity resulted in 1.81 kg (95% CI: −3.47, −0.16, *p* = 0.03) of GWG reduction in intervention groups. Healthy eating had a larger effect size (−5.77 kg, 95% CI −9.34, −2.21, *p* = 0.02) than combined healthy eating and physical activity (−0.82 kg, 95% CI −1.28, −0.36, *p* = 0.0005) in limiting GWG. They concluded that healthy eating with calorie and macronutrient goals are especially effective in limiting excessive GWG among pregnant overweight and obese women. The outcome of the neonates was not studied [20].

One Cochrane review evaluated the effectiveness and safety of diet or exercise, or both, interventions for preventing excessive weight gain in pregnant women of any BMI [21]. Diet or exercise, or both, interventions resulted in an average reduction of excessive GWG, usually defined according to prevailing IOM guidelines, of 20% in favor of the intervention group (RR 0.80, 95% CI 0.73, 0.87). There was no difference the between intervention and control groups for preterm birth (average RR 0.91, 95% CI 0.68, 1.22), for macrosomia or LGA (respectively, RR 0.93, 95% CI 0.86, 1.02 and RR 0.92, 95% CI 0.80, 1.05). Moreover, the risk for shoulder dystocia, birth trauma, hypoglycemia and hyperbilirubinemia was not different between the groups. Neonatal respiratory distress syndrome was the only neonatal outcome with significant risk reduction in the intervention groups (RR 0.47, 95% CI 0.26, 0.85) (more than 2000 participants with diet and exercise counselling interventions conducted in overweight and obese women).

The International Weight Management in Pregnancy (i-WIP) Collaborative Group published a meta-analysis of individual participant data from RCT to synthetize the evidence on effects of interventions based on diet and physical activity during pregnancy [22]. Based on IPD meta-analysis, diet or physical activity based interventions, or a combination of both resulted in significantly less GWG compared with control (−0.70 kg, 95% CI −0.92, −0.48 kg), but the risk of GDM was not significantly reduced (OR 0.89, 95% CI 0.72 to 1.10). No strong evidence was found to suggest that interventions had an effect on preterm birth or individual adverse offspring outcomes (stillbirth, LGA, SGA and admission to a neonatal intensive care unit). These results were not different according to maternal BMI. All three individual interventions (diet, physical activity and mixed) had a similar effect on reducing GWG by an average of 0.7 kg.

Ruchat et al. examined the relationship between prenatal exercise and GWG. They showed that exercise-only interventions compared with no exercise decreased total GWG (−0.9 kg, 95% CI −1.23, −0.57 kg) and also the risk of excessive GWG by about 30% (OR 0.68, 95% CI 0.57, 0.80) [23].

None of the studies to control GWG reported strong data on long-term outcomes in offspring.

The results of these studies are summarized in Table 2.

### 3.2. Pre-Pregnancy Weight Loss: Bariatric Surgery

Bariatric surgery (BS) is thought to be an effective intervention to sustain weight loss and is increasingly being used as an effective treatment for obesity. BS procedures are generally categorized into three groups. Restrictive procedures (laparoscopic adjustable gastric banding and sleeve gastrectomy) lead to weight loss by reducing gastric capacity which in turn restricts energy intake. Malabsorptive procedure (biliopancreatic diversion) leads to weight loss by restricting absorption of nutrients. Malabsorptive and restrictive procedures (Roux-en-Y gastric bypass) reduces stomach capacity, thereby causing malabsorption and a certain degree of restriction of food intake.

There are two meta-analyses under the scope of this paper that evaluated the effects of BS on pregnancy and neonatal outcomes. We also considered the meta-analysis of Galazis et al. that was published in October 2014.

Galazis et al. included 17 non-randomized cohorts or case-control studies that evaluated a total of 166,134 participants, which included 5361 women who underwent BS and 160,773 controls. They showed that compared to controls, in the BS group, there was a lower incidence of GDM (OR 0.47, 95% CI 0.40, 0.56; *p* < 0.001) and LGA (OR 0.46, 95% CI 0.34, 0.62; *p* < 0.001) and a higher incidence of SGA (OR 1.93, 95% CI 1.52, 2.44; *p* < 0.001), preterm birth (OR 1.31, 95% CI 1.08, 1.58; *p* = 0.006), and admission for neonatal intensive care (OR 1.33, 95% CI 1.02, 1.72; *p* = 0.03). There was no significant difference in the incidence of perinatal mortality [24].

Yi et al. conducted a meta-analysis of 11 cohort studies and 4178 obese women who had undergone BS and 16,016 women who had not. Among the women who had undergone BS, there was an apparent reduction in average BMI from 40–50 to 32–35 kg/m^2^. BS improved pregnancy outcomes with a lower risk of GDM (OR 0.31; 95% CI 0.15, 0.65) and macrosomia (OR 0.40; 95% CI 0.24, 0.67) but a higher risk of SGA (OR 2.16; 95% CI 1.28, 3.66) [25]. The risk of preterm birth was not modified by BS in this analysis and GWG was not studied.

A larger meta-analysis, including 20 cohort studies and approximately 2.8 million subjects, 8364 of whom had BS, was published by Kwong et al. [26]. When compared with control subjects who were matched for pre-surgery BMI, patients who underwent BS showed reduced rates of GDM (OR 0.20; 95% CI 0.11, 0.37) and of LGA (OR 0.31; 95% CI 0.17, 0.59). However, there was an increased risk for SGA (OR 2.16 95% CI 1.34–3.48), and for preterm birth (1.35, 95% CI 1.02, 1.79). There was no difference in rates of malformations, neonatal death and neonatal intensive care unit admissions.

Compared with restrictive surgeries, malabsorptive surgeries resulted in a greater increase in SGA (OR 2.39; 95% CI, 1.94, 2.94 versus OR 1.38; 95% CI 0.90, 2.10; *p* = 0.023) and a greater decrease in LGA (OR 0.28; 95% CI 0.22, 0.36 versus OR 0.50; 95% CI, 0.35, 0.73; *p* < 0.012). The authors were not able to account for the amount of weight loss relative to the pre-surgical weight, nor to analyze GWG.

Data on long-term outcomes such as the effects of BS on metabolic risk in the offspring into childhood and adulthood are inconsistent [27].

### 3.3. Lifestyle Interventions in Mothers with GDM to Improve Neonatal Heath

#### 3.3.1. Lifestyle Interventions: Diet, Exercise and Others

A review from Brown et al. suggested that for women diagnosed with GDM, and receiving lifestyle interventions (two or more interventions including dietary advice, physical activity, education or self-monitoring of blood glucose), there was a benefit for their neonates mainly due to reduced fetal growth. In the neonates, lifestyle interventions were associated with a decreased risk of being LGA (RR 0.60, 95% CI 0.50, 0.7), a reduction of macrosomia (RR 0.64, 95% CI 0.48, 0.87), a lower birth weight (−109.64 g, 95% CI −149.77, −69.51) and a decreased neonatal fat mass (−37.30 g, 95% CI −63.97, −10.63), compared with the control group. There was also a reduced risk of being born preterm (< 37 weeks’) (RR 0.71, 95% CI 0.53, 0.96) and of shoulder dystocia (RR 0.38, 95% CI 0.21, 0.66), but no difference was found for other adverse neonatal outcomes [28]. Lifestyle interventions were also associated with a decrease in weight gain in pregnancy (−1.30 kg, 95% CI −2.26, −0.35) and an increased use of additional pharmacological therapies. In this meta-analysis, there was a wide variety of interventions including exercise, diet, self-monitoring of blood glucose and education. This makes it hard to determine which of the interventions is more effective, especially since most of the interventions included a dietary component.

Follow-up into childhood was poorly reported with only three of the 15 included trials contributing data which could not be combined in a meta-analysis [29,30,31]. There was no difference between groups in infancy at ages 4 to 11 years, for BMI greater or equal to the 85th percentile and no difference in dyslipidemia or blood pressure.

#### 3.3.2. Lifestyle Intervention: Diet Alone

To date, a wide range of dietary advice interventions have been investigated in women with GDM, including low glycemic index (GI) diets, energy restricted diets, increase or decrease in carbohydrates, and modifications of fat or protein quality or quantity. Four systematic reviews analyzed the effect of the different diets on maternal and neonatal outcomes. The main results concerning the most commonly used diets are presented in Table 3 [32,33,34,35].

The energy-restricted diets may have different designs; for example, a calorie restricted diet of 35 kcal per kg ideal body weight per day or a restricted daily energy intake to 1200 kcal. The GI is used to estimate the in vivo blood glucose response to the intake of a food item, relative to that of a carbohydrate reference. The GI ranks food items on a scale of 0 to 100, with food items with higher GI values contributing to a greater increase in blood glucose. Low GI diets are based on foods with GI less than 55, producing a lower postprandial glucose elevation. Dietary Approaches to Stop Hypertension (DASH) diet is a diet rich in fruits, vegetables, whole grains and low-fat dairy products, and low in saturated fats, cholesterol, refined grains and sweets. In the low carbohydrate diet, the daily total energy intake from carbohydrates is 40% to 45% compared 55% to 60% for the control group.

When considering all diets, Yamamoto et al. showed that modified dietary interventions decreased maternal glycemic values and were significantly associated with significantly lower needs for medication treatment. This result was mainly due to low GI diets that showed a larger decrease in fasting, postprandial, and post-breakfast glucose compared with control diets [35]. The three analyses from Viana et al., Wein et al. and Han et al. reported a lower need for medication with low GI diets. The others dietary advice interventions reported in Table 3 had not effect on maternal medication need, excepted DASH diet. Only low carbohydrates and DASH diets were associated with a significant reduction in GWG in the analysis from Han et al. [34].

When considering all diets together, modified dietary interventions were significantly associated with less macrosomia and lower infant birth weight [35]. However, results concerning the effect of low GI diets on fetal growth are controversial. Viana et al. found that diet with low GI significantly reduced birth weight but not the risk of having macrosomia. Wei et al. found that low GI diets reduced the risk of macrosomia. Additionally, a subgroup analysis showed that low GI diets with increased dietary fiber reduced the risk of macrosomia beyond that of a low GI diet alone. Han et al. and Yamamoto et al. found no difference for LGA, macrosomia and birth weight with low GI diet. Energy restricted diets and low carbohydrate diets showed no benefit on fetal growth. Only DASH diet was associated with reduced relative risk of macrosomia, but not of LGA, and reduced birth weight.

None of the dietary advice interventions studied were associated with increased risk of neonatal hypoglycemia. In addition, there were significantly more neonates with hypocalcaemia born to women in the energy-restricted diet group compared with the no energy restriction group (RR 1.36, 95% CI 1.00, 1.86) [34]. None of the dietary interventions reported in Table 3 was associated with increased risk of being preterm or SGA [33,34].

In the Cochrane review, Han et al. reported that perinatal mortality and stillbirth was studied in only one trial comparing low versus high carbohydrate diet and did not find a difference between the groups. Likewise, there were no differences between groups for shoulder dystocia and hyperbilirubinemia in trials comparing low energy-restricted diet versus no restriction [34].

None of these studies reported strong data on long-term outcome in offspring.

#### 3.3.3. Lifestyle Intervention: Exercise-Only

In a Cochrane review, including 11 randomized trials, involving 638 women, Brown et al. specifically evaluated the effects of exercise interventions (any type of exercise program targeted at women with GDM at any stage of pregnancy) for improving maternal and fetal outcomes in women with GDM [36]. Exercise was associated with a reduced fasting and post-prandial blood glucose concentration compared with control, but there was no difference between groups for weight gain in pregnancy. There was no difference between groups for the composite outcome of mortality and morbidity (variously defined by trials, e.g., perinatal or infant death, shoulder dystocia, bone fracture or nerve palsy) (RR 0.56, 95% CI 0.12, 2.61), for being born macrosomic (RR 0.69, 95% CI 0.35, 1.35, n = 296) or for neonatal hypoglycemia (RR 2.00, 95% CI 0.20, 20.04).

In a meta-analysis involving 3670 women, Davenport et al. reported a 39% reduction in the odds of having a macrosomic baby (OR 0.61, 95% CI 0.41, 0.92) in women who exercised during the prenatal period compared with women who did not exercise, without affecting the risk of growth-restricted, preterm or low birth weight babies. Prenatal exercise was not associated with infant weight, obesity or body fat [37].

## 4. Discussion

### 4.1. Summary of the Results

Overall, trials that evaluated the effect of lifestyle interventions to reduce the risk of GDM reported a decreased risk of 15 to 40% in GDM, with a greater effect of exercise than diet. They also reported a variable decrease in GWG from 1 kg to 4 kg, when it was evaluated. The effects on adverse neonatal outcomes were poorly studied, particularly very weak effects were reported on neonatal anthropometry.

Combined lifestyle interventions specifically designed to limit GWG reduced GWG by 1.6 kg in overweight and obese women. Other lifestyle interventions to reduce GWG in all pregnant women decreased GWG on average by 0.7 to 1 kg. Diets that control calories and macronutrients had the most important effect on weight gain. Most of these interventions did not reduce the risk of GDM, excepted measures based on exercise which reduced this risk by about 35%–40%. The effects on adverse neonatal outcomes were limited. In particular, no effect was reported on growth at birth. Only two studies reported a reduced risk in preterm birth and one study reported a reduced risk of respiratory distress syndrome in infants of overweight and obese women.

BS before pregnancy decreased the risk of GDM by 70% to 80% and the risk of LGA by 60% to 70%. The risks for adverse neonatal outcomes were not modified by BS, except for a 2-fold increase in SGA and an increase of about 30% in preterm birth.

Combined lifestyle interventions in pregnant women with GDM significantly reduced fetal growth and neonatal fat mass. When all diets were considered together, they reduced the risk of macrosomia and birth weight. But, individually, specific diets had controversial or no effect on fetal growth, except the DASH diet, which reduced the risk of macrosomia and birth weight. Exercise alone during pregnancy with GDM had no effect on the risk of macrosomia, but prenatal exercise reduced it by 40%. Altogether, lifestyle interventions during pregnancy with GDM reduced the risk of preterm birth and shoulder dystocia, but individually, diets or exercise alone had no effect on neonatal adverse outcomes. Furthermore, energy restricted diet was associated with an increased risk of neonatal hypocalcaemia.

### 4.2. Strengths and Limitations of the Method

Many and various lifestyle interventions were evaluated during pregnancy and a large number of RCTs have been conducted. Individual studies have a limited ability to show effects on outcomes due to concomitant interventions and population size, and because outcomes are rare and difficult to identify. This is why we chose to work on the basis of meta-analyses which allow to pool the results of individual studies, especially since a large number of patients is required to conclude that an effect has occurred. Nonetheless, an additional limit is the heterogeneity of data. In particular, various diagnostic criteria for GDM were used in the different studies, and the outcomes evaluated were not always the same, were not uniformly standardized, or even not available. Another potential weakness is the small number of studies included in each dietary intervention category. Moreover, it was difficult to take into consideration the design of individual program intervention, the time of pregnancy when it was initiated and applied, and the compliance of patients to these interventions.

The limitation of aggregate data in meta-analysis can be addressed by meta-analyses of individual participant data, where the raw patient-level data are obtained and synthesized across trials. We have identified two such studies, but they provided limited information on the outcome of the newborn [19,22].

### 4.3. Effectiveness of Interventions in Pregnant Women

Lifestyle interventions can significantly reduce the risk of GDM with clinical relevance, but the limitation of GWG is low from a clinical standpoint. In addition, the benefits for the neonates and the offspring are limited, with a more significant impact on anthropometry of the neonates, if any. Among dietary intervention, low GI and DASH diets seem to be the most efficient.

Exercise alone or associated with diet, before or during pregnancy, significantly reduces the risk of GDM and GWG and improves fetal growth. Nevertheless, it is necessary to define precisely the type, duration and frequency of exercise that may have the greatest impact. The earlier the program is initiated during pregnancy, the better the results are.

Finally, the effects of lifestyle interventions on various groups of women based on BMI category, age, parity and risk status in pregnancy are difficult to assess or are not known.

### 4.4. Why Haven Lifestyle Interventions Not Achieved Better Outcomes for the Offspring?

Overall, the benefits of maternal lifestyle interventions are disappointing for the offspring. They do not significantly modify birth weight and have a limited impact on neonatal outcomes.

We hypothesize that the outcomes assessed are not entirely appropriate. Indeed, the measure of neonatal adiposity is probably more relevant than birth weight in assessing the effect of an intervention. In a recent RCT that included 334 neonates, counselling pregnant women with a BMI ≥29 kg/m^2^ on both healthy eating and physical activity resulted in a significant reduction of neonatal adiposity (−63 g for fat mass, and −1.2% for fat percentage), although birth weight was not different between groups. Interestingly, these changes were not mediated by GWG alone, but a reduction in sedentary time (notably sitting time) drove the effect of the intervention on neonatal adiposity [38].

The majority of studies focus on interventions initiated after pregnancy was diagnosed. However, many physio pathological arguments demonstrate that these interventions are initiated too late to show an effect on the mother and the newborn. Indeed, Catalano et al. showed that pre-gravid women with normal glucose tolerance who developed GDM in late gestation had subclinical metabolic dysfunction prior to conception compared to women with normal glucose tolerance [39]. Their research also suggests that maternal pre-gravid and early pregnancy metabolic conditions associated with obesity, such as increased insulin resistance and inflammation, affect early placenta functions and genes expression. These alterations in placental functions occur in the first trimester of pregnancy before most intervention trials are initiated [40]. The placenta is at the interface of the maternal and fetal environment and its function plays a major role on the impact of maternal health on fetal development. Maternal diabetes and obesity lead to modifications to substrates transport and metabolism across the placenta. This contributes to adverse fetal outcomes, the most common being macrosomia [41]. This also explains why even in pregnant women with well controlled diabetes, macrosomia remains common. All these elements argue in favor of the initiation of lifestyle interventions prior to pregnancy in order to obtain significant effects in the neonate.

BS represents an extreme model of pre-gestational intervention, with often significant weight loss in mothers before pregnancy. Compared with mothers with the same pre-gestational BMI, BS is associated with a significantly decreased risk of macrosomia, but also with an increased risk of low birth weight. In addition, BS exposes pregnant women to the risk of nutritional deficiencies with poorly known effects in the newborn and the offspring [42]. This shows that pre-gestational weight loss may have positive effects in limiting fetal growth, but that it has to be dosed reasonably to avoid adverse effects.

In addition, the benefit of lifestyle interventions on long-term outcomes was poorly studied in the meta-analyses and the few results available are inconsistent. The long-term effect of maternal diabetes on offspring outcome is controversial. Some authors claimed that there is an association between maternal diabetes and offspring diabetes and obesity [43]. Others showed that the association of maternal glucose levels during pregnancy with childhood adiposity is generally attenuated after adjusting for maternal BMI [44,45]. Recently, large cohort studies reported new evidence between the association of GDM and offspring glucose metabolism and adiposity. The HAPO Follow-up Study (HAPO FUS) followed more than 4500 children ages 10 to 14 years of age and examined the associations between maternal glucose levels during pregnancy and childhood glucose metabolism and adiposity.

It showed that GDM was significantly and independently associated with childhood impaired glucose tolerance and with childhood adiposity [46,47]. In addition, in a large population-based cohort study (2,432,000 live born offspring), during up to 40 years of follow-up, GDM was associated with increased rates of early onset cardio-vascular disease in offspring, persisting from childhood through early adulthood [48]. However, there is little evidence suggesting that the usual treatment of GDM affects long-term outcomes in the offspring. In a follow-up study of children (ages 5–10) born to women enrolled in a multicenter trial with treatment (diet therapy and insulin if required) versus no treatment of mild GDM, no reduction in childhood obesity or metabolic dysfunction in the offspring of treated women was found, except for lower fasting glucose in female offspring only. It is thus thought that the effect of lifestyle interventions will have limited effects on the long-term outcome of the offspring or that it might be difficult to demonstrate.

## 5. Conclusions

Specific maternal, neonatal and offspring benefits of lifestyle interventions during pregnancy to prevent or improve GDM control or to limit GWG still require clarification. While ultimately healthy lifestyle is a matter of individual behavior change, individual interventions must extend beyond individual targeted initiatives to address societal and environmental factors and enable children, adolescents and women to have a healthier lifestyle in order to prevent obesity and related complications before pregnancy [49].

## Figures and Tables

**Table 1:** Effect of lifestyle interventions during pregnancy on the risk of gestational diabetes (GDM), on gestational weight gain (GWG) and the risk of adverse neonatal outcomes according to meta-analyses. RDS: respiratory distress syndrome. ND: not determined.

		Risk for GDM RR or OR [95% CI]	*p*	GWG Mean [SD]	*p*	Adverse Neonatal Outcomes
**Diet, exercise or both**	N					
Song 2016 [10]	11,487	0.82 [0.70, 0.95]	0.033	ND		ND
Guo 2019 [12]	15,745	0.77 [0.69, 0.87]	<10^−4^	ND		ND
Diet + exercise	N					
Song 2016 [10]	6047	0.85 [0.70, 1.03]	0.09	ND		ND
Shepherd 2017 [11]	8918	0.85 [0.71, 1.01]	0.07	−0.89 [−1.39, −0.40]	<10^−3^	Macrosomia: 0.63 [0.42, 0.94] Preterm birth: 0.80 [0.65, 0.98] RDS: 0.56 [0.33, 0.97]
Guo 2019 [12]	7024	0.86 [0.71, 1.04]	ND	ND		ND
Diet	N					
Song 2016 [10]	1279	0.80 [0.58, 1.10]	0.17	ND		ND
Tieu 2017 [13]	1279	0.60 [0.35, 1.04]	0.07	−4.70 [−8.07, −1.34]	0.01	None
Guo 2019 [12]	2838	0.75 [0.60, 0.95]	ND	ND		ND
Exercise	N					
Sanabrina-Martinez 2015 [14]	2873	0.69 [0.52, 0.91]	0.009	−1.14 [−1.50, −0.78]	<10^−3^	ND
Russo 2015 [15]	3401	0.72 [0.58, 0.91]	0.005	ND		ND
Song 2016 [10]	4161	0.77 [0.54, 1.09]	0.15	ND		ND
Davenport 2018 [17]	6934	0.62 [0.52, 0.75]	ND	ND		ND
Guo 2019 [12]	5883	0.70 [0.59, 0.84]	ND	ND		ND
Exercise in normal weight women	N					
Ming 2018 [16]	2981	0.58 [0.37, 0.90]	0.01	−1.61 [−1.99, −1.22]	<10^−2^	None

**Table 2:** Effects of lifestyle interventions on gestational weight gain (GWG) and the risk of gestational diabetes (GDM), and the risk of adverse neonatal outcomes according to meta-analyses. Only significant effects on adverse neonatal outcomes are reported in the table. RDS: respiratory distress syndrome. ND: not determined.

		GWG kg Mean [SD]	Risk for GDM OR or RR [95% CI]	Adverse Neonatal Outcomes OR or RR [95% CI]
**All lifestyle interventions**	N			
Peaceman 2018 * [19]	1150	−1.58 [−2.18, −0.99]	0.92 [0.61, 1.40]	Preterm birth < 28 wks: 0.48 [0.22, 0.95]
Diet, exercise or both	N			
Muktabhan 2015 [21]	11,444	Risk of excessive GWG RR [95% CI] 0.80 [0.73, 0.87]	ND	RDS in overweight/obese: 0.47 [0.26, 0.85]
i-WIP 2017 [22]	9320	−0.70 [−0.92, −0.48]	0.89 [0.72, 1.10]	None
Shieh 2018 * [20]	6920	−1.81 [−3.47, −0.16]	ND	ND
Diet	N			
i-WIP 2017 [22]	1168	−0.72 [−1.48, −0.04]	1.03 [0.30, 3.61]	Preterm birth: 0.28 [0.08, 0.96]
Shieh 2018 * [20]	719	−5.77 [−9.34, −2.21]	ND	ND
Exercise	N			
Sanabrina-Martinez 2015 [14]	2873	−1.14 [−1.50, −0.78]	0.69 [0.52, 0.91]	ND
i-WIP 2017 [22]	2915	−0.73 [−1.11, −0.34]	0.67 [0.46, 0.99]	None
Ruchat 2018 [23]	5819	−0.90 [−1.23, −0.57]	ND	ND
Diet + exercise	N			
i-WIP 2017 [22]	2981	−0.71 [−1.10, −0.31]	1.02 [0.79, 1.32]	None
Shieh 2018 * [20]	5853	−0.82 [−1.28, −0.36]	ND	ND

* only women with BMI ≥ 25 kg/m^2^.

**Table 3:** Effect of diet advice interventions in women with gestational diabetes on the risk of maternal medication use, on gestational weight gain (GWG), on the growth at birth and on the risk of neonatal hypoglycaemia according to meta-analyses. Values are in bold when the results were statistically significant; *p* values are indicated when they were reported in the reviews. LGA: Large for Gestational Age.

		MOTHER		NEONATE			
	N	Medication RR [95% CI]	GWG kg Mean [SD]	LGA RR [95% CI]	Macrosomia RR [95% CI]	Birth Weight g Mean [SD]	Hypoglycaemia RR [95% CI]
Viana 2014 [32]							
Low glycemic index	257	**0.77** [0.66, 0.99] **	−0.41 [−1.84, 1.02]	ND	0.48 [0.15, 1.56]	−161.9 [−246.4, −77.4] ¶	ND
Energy restriction diet	425	ND	ND	ND	1.00 [0.65, 1.55]	ND	1.01 [0.72, 1.43]
Low carbohydrates	182	1.06 [0.15, 1.56]	ND	ND	0.35 [0.06, 1.91]	ND	ND
Wei 2016 [33]							
Low glycemic index	302	0.67 [0.44–1.00] *	ND	1.38 [0.58, 3.32]	0.27 [0.10, 0.71] §	ND	ND
Han 2017 [34]							
Low glycemic index	224	0.82 [0.39, 1.74]	−0.47 [−2.18, 1.24]	0.71 [0.22, 2.34]	0.59 [0.16, 2.26]	−56.0 [−201.9, 89.9]	ND
Energy restriction diet	437	1.05 [0.47, 2.34]	+1.88 [−1.96, 5.72]	1.17 [0.65, 2.21]	0.99 [0.64, 1.53]	−107.0 [−240.3, 26.3]	1.06 [0.48, 2.32]
Low carbohydrates	182	1.02 [0.77, 1.37]	−0.90 [−1.60, −0.20]	0.51 [0.13, 1.95]	0.20 [0.02, 1.69]	+ 22.0 [−241.1, 285.1]	0.91 [0.39, 2.12]
DASH diet	136	0.28 [0.14, 0.53] **	−2.88 [−8.48, −2.71]	ND	0.10 [0.01, 0.73]	−581.3 [−790.3, −372.2]	ND
Yamamoto 2018 [35]							
All diet	1023	0.65 [0.47, 0.88]	ND	0.96 [0.63, 1.46]	0.49 [0.27, 0.88] **	−171.6 [−333.6, −7.6] **	ND
Low glycemic index		0.80 [0.55, 1.14]	ND	1.33 [0.54, 3.31]	0.46 [0.15, 1.46]	−54.2 [−179.0, 70.5]	ND
Energy restriction diet		1.05 [0.47, 2.34]	ND	1.17 [0.65, 2.12]	1.56 [0.61, 3.94]	194.0 [−42.6, 430.6]	ND
Low carbohydrates		1.00 [0.75, 1.34]	ND	0.51 [0.13, 1.95]	0.20 [0.02, 1.69]	57.7 [−164.9, 280.4]	ND

* *p* = 0.05; ** *p* < 0.05; § *p* ≤ 10^−2^; ¶ *p* ≤ 10^−3^.
